# The *NAC*-like gene *ANTHER INDEHISCENCE FACTOR* acts as a repressor that controls anther dehiscence by regulating genes in the jasmonate biosynthesis pathway in *Arabidopsis*

**DOI:** 10.1093/jxb/ert412

**Published:** 2013-12-09

**Authors:** Ching-Fang Shih, Wei-Han Hsu, Yan-Jhu Peng, Chang-Hsien Yang

**Affiliations:** ^1^Institute of Biotechnology, National Chung Hsing University, Taichung, Taiwan 40227 ROC; ^2^Agricultural Biotechnology Center, National Chung Hsing University, Taichung, Taiwan 40227 ROC

**Keywords:** Anther dehiscence, *ANTHER INDEHISCENCE FACTOR*, jasmonate signalling, *NAC*-like gene, repressor.

## Abstract

*ANTHER INDEHISCENCE FACTOR* (*AIF*), a *NAC*-like gene, was identified in *Arabidopsis.* In *AIF:GUS* flowers, β-glucuronidase (GUS) activity was detected in the anther, the upper parts of the filaments, and in the pollen of stage 7–9 young flower buds; GUS activity was reduced in mature flowers. Yellow fluorescent protein (YFP)+AIF-C fusion proteins, which lacked a transmembrane domain, accumulated in the nuclei of the *Arabidopsis* cells, whereas the YFP+AIF fusion proteins accumulated in the membrane and were absent in the nuclei. Further detection of a cleaved AIF protein in flowers revealed that AIF needs to be processed and released from the endoplasmic reticulum in order to function. The ectopic expression of *AIF-C* caused a male-sterile phenotype with indehiscent anthers throughout flower development in *Arabidopsis*. The presence of a repressor domain in AIF and the similar phenotype of indehiscent anthers in *AIF-C+SRDX* plants suggest that *AIF* acts as a repressor. The defect in anther dehiscence was due to the down-regulation of genes that participate in jasmonic acid (JA) biosynthesis, such as *DAD1/AOS/AOC3/OPR3*/*OPCL1*. The external application of JA rescued the anther indehiscence in *AIF-C* and *AIF-C+SRDX* flowers. In *AIF-C+VP16* plants, which are transgenic dominant-negative mutants in which *AIF* is converted to a potent activator via fusion to a VP16-AD motif, the anther dehiscence was promoted, and the expression of *DAD1/AOS/AOC3/OPR3*/*OPCL1* was up-regulated. Furthermore, the suppression of *AIF* through an antisense strategy resulted in a mutant phenotype similar to that observed in the *AIF-C+VP16* flowers. The present data suggest a role for *AIF* in controlling anther dehiscence by suppressing the expression of JA biosynthesis genes in *Arabidopsis*.

## Introduction

Anther dehiscence is an essential process in which mature pollen grains are released from the locules of the anther, thus enabling pollination. Although morphological changes in the anther during dehiscence have been clearly described (Goldberg *et al.*, 1993; Sanders *et al.*, 1999; Scott *et al.*, 2004), the molecular mechanisms controlling dehiscence remain relatively unknown.

Jasmonic acid (JA) has been thought to play an important role in regulating anther dehiscence (Sanders *et al.*, 2000; Zhao and Ma, 2000; Ishiguro *et al.*, 2001; Scott *et al.*, 2004; Ma, 2005). Mutations in genes that participate in JA biosynthesis cause a failure or delay in anther dehiscence and can result in male sterility. Examples of these genes include the *DEFECTIVE IN ANTHER DEHISCENCE 1* (*DAD1*) gene, which encodes a phospholipase A1 that catalyses the initial step of JA biosynthesis (Ishiguro *et al.*, 2001); *AOS*, a gene that encodes allene oxide synthase (Park *et al.*, 2002; von Malek *et al.*, 2002); *DEHISCENCE 1* (*DDE1*)/*OPR3*, which encodes the OPR protein 12-oxo-phytodienoic acid reductase in the JA synthesis pathway (Sanders *et al.*, 1999, 2000; Stintzi and Browse, 2000; Ma, 2005); and the triple mutation (*fad3 fad7 fad8*) of genes encoding fatty acid desaturases that catalyse the desaturation of linoleic acid to form linolenic acid (McConn and Browse, 1996). In addition, similar phenotypes have been observed in *coronatine insensitive 1* (*coi1*) mutants, which are insensitive to JA (Feys *et al.*, 1994; Xie *et al.*, 1998), indicating that JA is specifically required for anther dehiscence during anther development. JA has been thought to control dehiscence by regulating water transport in the filament and anther because mutants defective in JA synthesis have much less water loss in anther tissues than do non-mutants (Ishiguro *et al.*, 2001; Ma, 2005). However, only a few works have described a possible mechanism by which the regulation of JA activity is associated with anther dehiscence (Nagpal *et al.*, 2005; Ito *et al.*, 2007; Cheng *et al.*, 2009; Peng *et al.*, 2013). One of these works describes two auxin response factors, ARF6 and ARF8, in auxin signalling that are thought to play roles in regulating JA production (Nagpal *et al.*, 2005). In addition, auxin has been thought to control anther dehiscence by regulating both JA biosynthesis and endothecium lignification (Cecchetti *et al.*, 2013). Recently, a report described a RING-type E3 ligase that regulates anther dehiscence by activating the jasmonate biosynthetic pathway gene *DAD1* (Peng *et al.*, 2013). However, the precise molecular mechanisms regulating JA activity during anther dehiscence are still to be elucidated.

NAC is a domain that is named for the three genes first described to contain the domain: *NO APICAL MERISTEM* (*NAM*) of petunia and *ATAF1*/*2* and *CUP-SHAPED COTYLEDON 2* (*CUC2*) of *Arabidopsis* (Souer *et al.*, 1996; Aida *et al.*, 1997). The proteins that are encoded by *NAC*-like genes contain a conserved 150 amino acid NAC domain at the N-terminus in addition to a C-terminal domain that is diverse in both length and amino acid sequence (Souer *et al.*, 1996). *NAC*-like genes show no sequence homology to any other characterized proteins and are plant specific (Riechmann *et al.*, 2000). Their functions include an involvement in the development of the shoot apical meristem (Souer *et al.*, 1996; Aida *et al.*, 1997, 2002; Takada *et al.*, 2001; Baurle and Laux, 2003), the control of cell expansion in specific flower organs (Sablowski and Meyerowitz, 1998), and the regulation of lateral root formation (Xie *et al.*, 2000). A group of membrane-bound NAC transcription factors (designated NTLs) has been reported to be closely linked to plant responses to environmental stresses (Kim *et al.*, 2007, 2008). Additionally, the induction of *NAC* gene expression by drought, high salinity, and abscisic acid has been reported (Tran *et al.*, 2004; He *et al.*, 2005). Furthermore, the functions of *NAC*-like genes include the regulation of senescence (Guo and Gan, 2006; Uauy *et al.*, 2006), secondary wall biosynthesis in fibres (Zhong *et al.*, 2006), and xylem differentiation (Kubo *et al.*, 2005; Zhao *et al.*, 2005). However, the functions of a large number of *NAC*-like genes remain unknown.

To explore further the functions of *NAC*-like genes, additional genes must be characterized. For this purpose, one *Arabidopsis NAC*-like gene, *ANTHER INDEHISCENCE FACTOR* (*AIF*), was characterized and functionally analysed using SRDX, VP16-AD, and antisense strategies in this study. The findings reveal a repressor role for *AIF* in preventing anther dehiscence during stamen development by suppressing genes that participate in JA biosynthesis.

## Materials and methods

### Plant materials and growth conditions

Seeds for *Arabidopsis* were sterilized and placed on agar plates containing 1/2 Murashige and Skoog medium (Murashige and Skoog, 1962) at 4 °C for 2 d. The seedlings were then grown in growth chambers under long-day conditions (16h light/8h dark) at 22 °C for 10 d before being transplanted to soil. The light intensity of the growth chambers was 150 μE m^–2^ s^–1^.

### Cloning of *Arabidopsis AIF* cDNA

*Arabidopsis AIF* (At3g10500), containing six exons and five introns, was identified on chromosome 3. cDNA containing an open reading frame of *AIF* was amplified by reverse transcription–PCR (RT–PCR) using the 5′ primer, F1-5 (AtNAC-3-1), and the 3′ primer, F1-3 (AtNAC-3-2). cDNA truncated with the C-terminal region of *AIF* (*AIF-C*) was amplified by RT–PCR using the 5′ primer, F1-C5 (AtNACL3 5′), and the 3′ primer, F1-C3 (NACL3 3′∆C-ter). All of the primers contained the generated *Xba*I recognition site (5′-TCTAGA-3′) to facilitate the cloning of *AIF* cDNA. Sequences for the primers are listed in Supplementary Table S1 available at *JXB* online.

An *Xba*I fragment containing the cDNA truncated with the C-terminal region for the *AIF* gene was cloned into the linker region in binary vector pBImGFP3 (CHY Lab, Taichung, Taiwan) under the control of the *Cauliflower mosaic virus* (CaMV) 2×35S-Ω promoter (*35S:AIF-C*) and used for further plant transformation.

### *AIF:GUS* fusion construct

For the *AIF*:*GUS* (β-glucuronidase) construct, the *AIF* promoter (2.56kb) was obtained by PCR amplification from the genomic DNA using the pAtNACL3 5′*Bam*HI and pAtNACL3 3′*Bam*HI primers and then cloned into pGEMT easy vector (Promega, Madison, WI, USA). The full-length promoter for *AIF* (2.56kb) was then subcloned into the linker region before the GUS coding region in binary vector pBI101 (Clontech, Palo Alto, CA, USA). The primers contained the generated *Bam*HI (5′-GGATCC-3′) recognition site to facilitate the cloning of the promoter. Sequences for the primers are listed in Supplementary Table S1 at *JXB* online.

### Construction of the *AIF-C+SRDX* construct

To clone the DNA sequence encoding SRDX (LDLDLELRLGFA*), a PCR fragment was amplified, using the mGFP5 sequence as a template, with two rounds of PCR with the primers SRDX-for/mGFP-revII and SRDX-forII/mGFP-revII. The primers contained the *Kpn*I and *Sac*I recognition sites to facilitate the cloning of SRDX. The SRDX-for and SRDX-forII primers contained the overlapping sequence that encoded LDLDLELRLGFA and a stop codon (TGA) in the end of the SRDX sequence. The PCR fragment was inserted between the *Kpn*I and *Sac*I sites in the binary vector, pEpyon-32K (CHY Lab), from which the whole mGFP5 (*Kpn*I–*Sac*I fragment) was removed to produce a SRDX fusion gene driven by the 2×35-Ω promoter, and was named pEpyon-3aK. For the *AIF-C+SRDX* construct, the cDNA for *AIF-C* was obtained by PCR amplification using the AtNACL3 5′-2 and AtNACL3 (delC) 3′ primers that contained the *Xba*I recognition site to facilitate the cloning of the cDNA. The 0.9kb fragment containing the cDNA for *AIF-C* was cloned into the pEpyon-3aK plasmid upstream of the SRDX sequence, under the control of the CaMV 35S promoter, and it was then used for plant transformation. The sequences for the primers are listed in Supplementary Table S1 at *JXB* online.

### Construction of the *AIF-C+VP16* construct

To clone the DNA sequence encoding the VP16-AD domain that included an 11 amino acid activation sequence (DALDDFDLDML), DNA was amplified using the plasmid pYESTrp3 (Invitrogen) as the template using two rounds of PCR with the primers VP16-for/VP16-rev and VP16-forII/VP16-rev. The primers contained the *Kpn*I and *Sac*I recognition sites to facilitate the cloning of VP16-AD. The *Kpn*I–*Sac*I VP16-AD fragment contained 78 amino acids, including DALDDFDLDML and a stop codon (TGA) at the end of the VP16-AD sequence. This *Kpn*I–*Sac*I VP16-AD fragment was inserted between the *Kpn*I and *Sac*I sites in the binary vector, pEpyon-22K, from which the entire mGFP5 (*Kpn*I–*Sac*I fragment) was removed to produce a VP16-AD fusion gene driven by the 1×35-Ω promoter and named pEpyon-2bK. For the *AIF-C+VP16* construct, the cDNA for *AIF-C* was obtained by PCR amplification using the AtNACL3 5′-2 and AtNACL3 (delC) 3′ primers that contained the *Xba*I recognition sites to facilitate the cloning of the cDNA. The PCR fragment containing the *AIF-C* was cloned into the pEpyon-2bK plasmid, in front of the VP16-AD sequence and under the control of the CaMV 35S promoter, and it was then used for plant transformation. The sequences for the primers are listed in Supplementary Table S1 at *JXB* online.

### Real-time PCR analysis

For real-time quantitative RT–PCR, the reaction was performed on an MJ Opticon system (MJ Research, Waltham, MA, USA) using SYBR Green Real-Time PCR Master Mix (TOYOBO Co., Ltd.). The amplification conditions were 95 °C for 10min, followed by 40 cycles of amplification (95 °C for 15 s, 58 °C for 15 s, and 72 °C for 30 s, followed by plate reading) and melting (50–95 °C with plate readings every 1 °C). The sequences for the primers that were used for the real-time quantitative RT–PCR for *AIF*, *At5g04410*, *DAD1*, *LOX1, LOX2*, *LOX3*, *AOS*, *AOC1*, *AOC2*, *AOC3*, *AOC4*, *OPR3*, and *OPCL1* are listed in Supplementary Table S1 at *JXB* online. The *Arabidopsis* housekeeping gene *UBQ10* was used as a normalization control with the primers RT-UBQ10-F and RT-UBQ10-4-2. All of the experiments were repeated at least twice for reproducibility. The data were analysed using Gene Expression Macro software (version 1.1, Bio-Rad) according to the manufacturer’s instructions. The ‘delta–delta method’ formula 2^–[ΔCP sample–ΔCP control]^, where 2 represents perfect PCR efficiency, was used to calculate the relative expression of the genes. To calculate the statistical significance, unpaired *t*-tests were used.

### Western blot analysis

Total proteins were isolated from flowers of wild-type plants as described previously (Jang *et al.*, 2005). For western blot analysis, proteins were separated by 10% SDS–PAGE and transferred to polyvinylidene difluoride (PVDF) membranes. AIF-specific antibody was used to detect various forms of peptides for AIF in immunoblot analyses.

### Plant transformation and transgenic plant analysis

Constructs made in this study were introduced into *Agrobacterium tumefaciens* strain GV3101 and transformed into *Arabidopsis* plants using the floral dip method as described elsewhere (Clough and Bent, 1998). Transformants that survived in the medium containing kanamycin (50 μg ml^–1^) were further verified by RT–PCR analysis.

### Histochemical GUS assay

Histochemical staining was performed under the standard method described previously (Jefferson *et al.*, 1987; Chou *et al.*, 2001).

### Alexander’s staining

For pollen analysis, the pollen grains were mounted with Alexander’s stain as previously described (Alexander, 1969).

### Scanning electron microscopy (SEM)

SEM was performed according to the method of Hsu and Yang (2002), Tzeng and Yang (2001), and Chang *et al.* (2010). Various floral organs were fixed in 2% glutaraldehyde in 25mM sodium phosphate buffer (pH 6.8) at 4 °C overnight. After dehydration in a graded ethanol series, specimens were critical-point dried in liquid CO_2_. The dried materials were mounted and coated with gold–palladium in a JBS sputter-coater (model 5150). Specimens were examined with a Field emission scanning electron microscope (JEOL JSM-6700F, Japan) with an accelerating voltage of 15kV.

### *Arabidopsis* mesophyll protoplast isolation and transient expression assay

Protoplast isolation and transient expression assays were performed according to the method described previously (Yoo *et al.*, 2007). For the *35S:YFP+AIF* and *35S:YFP+AIF-C* construct, cDNA containing an open reading frame of *AIF* was amplified by RT–PCR using the 5′ primer AtNACL3 5′X*ma*I and the 3′ primer AtNACL3 3′X*ma*I (*AIF*); and the 5′ primer AtNACL3 5′X*ma*I and the 3′ primer AtNACL3(-C) 3′X*ma*I (*AIF-C*). The primers contained the generated *Xma*I recognition site (5′-CCCGGG-3′) to facilitate the cloning of the cDNAs. The *Xma*I fragments containing the cDNA for *AIF* and *AIF-C* were cloned into the linker region after the yellow fluorescent protein (YFP) coding region in pWEN25 to generate *35S:YFP+AIF* and *35S:YFP+AIF-C* under the control of the CaMV 35S promoter. The construct was used for further protoplast PEG (polyethylene glycol) transfection.

For fluorescence microscopy, transfection protoplasts were cultured for 8h at room temperature under light. Visualization was performed using an Olympus BX51 System (Tokyo, Japan). After excitation by 490–500nm laser, the YFP fluorescence was collected at 515–560nm.

### Lignin staining

For lignin analysis, fresh anthers were stained with 0.01% auramine O (Pesquet *et al.*, 2005) and observed with a confocal microscope (Olympus FV1000). The lignified cells were observed under 488nm excitation/510–560nm emission.

### Application of methyl jasmonate (MeJA)

All opened flowers (after stage 14) were removed from the inflorescence, and the remaining flower bud clusters were dipped into 1000 μM MeJA (Sigma) dissolved in 0.05% aqueous Tween-20.

## Results

### Isolation of *AIF* cDNA from *Arabidopsis*

**Fig. 1.**
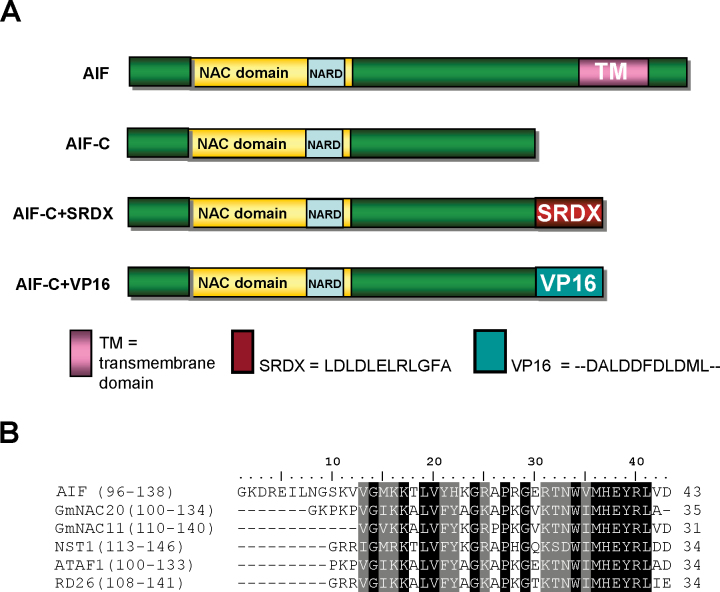
The protein structure of AIF. (A) The AIF protein contains a conserved NAC domain in the N-terminus, a conserved NARD domain for the repression of transcriptional activation in the end of the NAC domain, and a transmembrane domain (TM) in the C-terminus of the protein. In AIF-C, the TM was deleted. In AIF-C+SRDX, an SRDX domain that contained the 12 amino acid repressor sequence (LDLDLELRLGFA) was fused to AIF-C. In AIF-C+VP16, an activation domain, VP16-AD, which included the 11 amino acid activation sequence (DALDDFDLDML), was fused to AIF-C. (B) The conserved NARD domain identified in AIF and several other NAC-like proteins. (This figure is available in colour at *JXB* online.)

One *Arabidopsis NAC-like* gene, *ANTHER INDEHISCENCE FACTOR* (*AIF*) (At3g10500), was cloned and analysed. *AIF* contains six exons and five introns (Supplementary Fig. S1A at *JXB* online) and encodes a protein of 549 amino acids (Supplementary Fig. S1B). The AIF protein contains a conserved NAC domain with the five (A–E) putative subdomains (Supplementary Fig. S1B) that have been identified in the N-termini of most NAC-like proteins (Aida *et al.*, 1997; Kikuchi *et al.*, 2000). When the sequence of the AIF protein was further analysed, a conserved NAC repression domain (NARD), which represses transcriptional activation (Hao *et al.*, 2010), was identified in the end of the NAC domain (Fig. 1A, B; Supplementary S1B). The AIF protein showed 68% identity and 78% similarity to the most closely related NAC-like protein, At5g04410 (Supplementary Figs S1B, S2). In their NAC domains, 95% of the amino acids are identical (Supplementary Fig. S1B).

### Detection of *AIF* expression by analysing *AIF:GUS* transgenic *Arabidopsis*

**Fig. 2.**
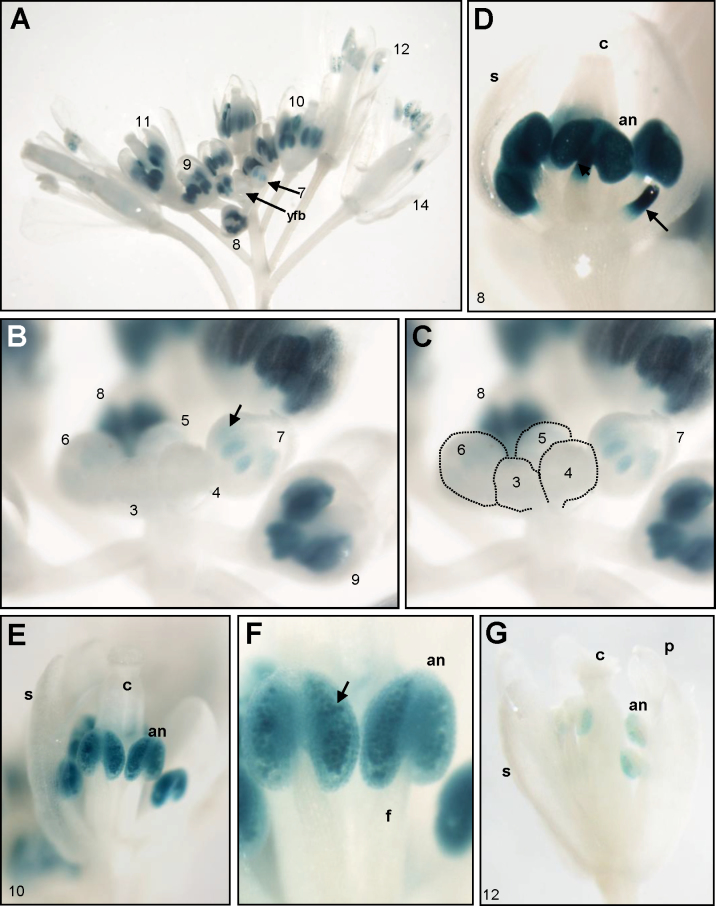
GUS staining patterns in *AIF:GUS* transgenic *Arabidopsis*. (A) GUS activity was specifically detected in the anthers of *AIF:GUS* floral buds (stages 7–11) and was absent in the young floral buds (yfb) before stages 5–6. The number indicates the stage of flower development. (B) Close-up of young floral buds at stages 3–9. GUS activity started to be detected in anthers (arrowed) of floral buds at stage 7 and was strongly expressed at stages 8–9. GUS was absent in the young floral buds before stages 5–6. (C) The corresponding young floral buds at stages 3–6 in (B) are indicated by dashed circles. (D) Close-up of a floral bud at stage 8. Strong GUS activity was detected only in anthers (an) and in the upper parts of filaments (arrowed). (E and F) Close-up of a floral bud (E) and anther (F) at stage 10. Strong GUS activity was detected in anthers (an) and pollen (arrowed). (G) GUS staining was weak in anthers (an) and pollen of an *AIF:GUS* mature flower at stage 12. c, carpel; s, sepal; p, petal; f, filament.

To investigate the expression pattern of the *AIF* gene, a construct (*AIF:GUS*) was generated and transformed into *Arabidopsis*, and >20 independent *AIF:GUS* plants were obtained. In the *AIF:GUS* transgenic seedlings and plants, GUS staining was only detected in the primordia of the emerged true leaves and was absent in the mature leaves. During flower development, GUS activity was not detected before stage 5–6 of flower budding (Fig. 2A–C). Strong GUS activity was detected only in the anthers and the upper parts of the stamen filaments after stage 7–9 of flower budding (Fig. 2A–D). In stage 10 flower buds, GUS activity in the filaments was absent but was continually detected in the anthers and pollen grains (Fig. 2A, E, F). The GUS activity in the anthers and pollen grains was greatly diminished in mature flowers after stage 12 (Fig. 2A, G). The specification of the flower developmental stages was as described by Smyth *et al.* (1990). The pattern of GUS expression during stamen development that was obtained in this study was in agreement with the *Arabidopsis* eFP browser data (Schmid *et al.*, 2005; Winter *et al.*, 2007) (http://www.bar.utoronto.ca/efp/cgi-bin/efpWeb.cgi), which report that *AIF* is detected in the stamens of young flowers and is barely expressed in mature flowers.

### AIF needs to be processed and released from the ER to perform its function

**Fig. 3.**
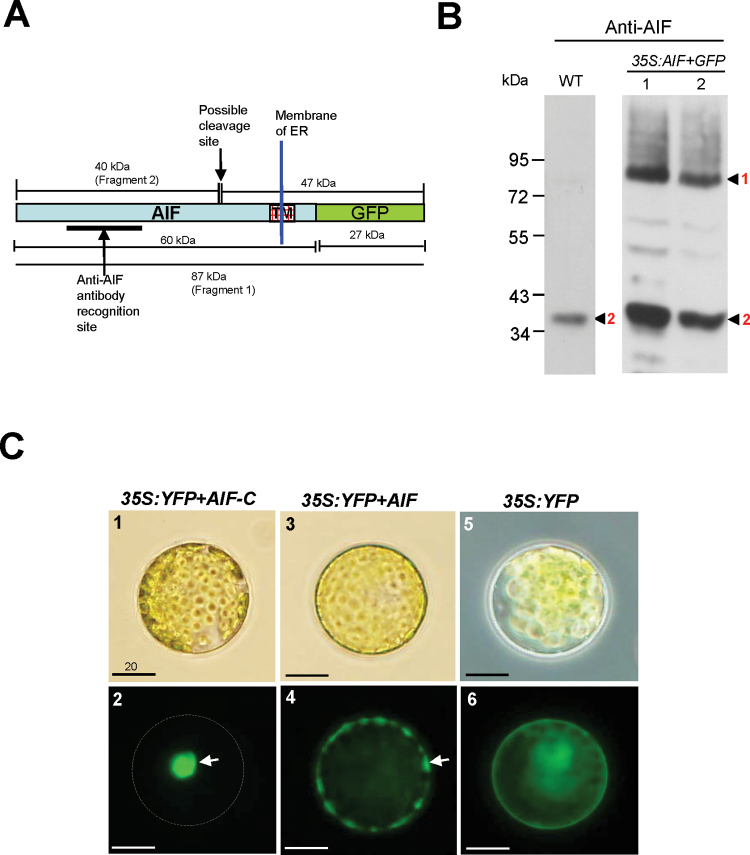
Detection of AIF proteins in flowers and the localization of AIF proteins in *Arabidopsis* protoplasts. (A) The protein structure of an uncleaved 87kDa AIF+GFP (Fragment 1) that contains the fusion of the proteins AIF (60kDa) and GFP (27kDa). Two peptides, 40kDa (Fragment 2) and 47kDa, will be produced after the AIF+GFP protein is processed, and the 40kDa Fragment 2 (AIF*-*C) will be released from the ER. The anti-AIF antibody recognition site and the possible cleavage site for AIF are indicated by arrows. The transmembrane domain (TM) in the C-terminus of the AIF protein is boxed. (B) The detection by western blot analysis of various forms of peptides for cleaved and uncleaved AIF+GFP in flowers. In wild-type flowers, only a cleaved 40kDa AIF*-*C band (Fragment 2) was detected by the anti-AIF antibody. In *35S:AIF+GFP* flowers, a cleaved 40kDa AIF*-*C band (Fragment 2) and an uncleaved 87kDa AIF+GFP band (Fragment 1) were detected by the anti-AIF antibody. (C) *Arabidopsis* protoplasts transfected with *35S:YFP+AIF-C* (C-1), *35S:YFP+AIF* (C-3), and *35S:YFP* (C-5). YFP+AIF-C (C-2), YFP+AIF (C-4), and YFP (C-6) fluorescence images of the transfected protoplasts from C-1, C-3, and C-5, respectively. The YFP+AIF-C fusion proteins accumulate in the nucleus (arrow) of the cell, and the corresponding cell membrane is indicated by the dashed circle (C-2). The YFP+AIF fusion proteins accumulate in the membrane (arrow) and are absent from the nucleus (C-4). The YFP proteins are dispersed in the cytoplasm (C-6). Bar=20 μm.

Kim *et al.* (2006) reported that a group of membrane-bound NAC transcription factors (designated NTLs) are transported into the nucleus to regulate the expression of downstream genes after release from the membranes. Because AIF is predicted to be a member of the NTL family, which contains a transmembrane domain in the C-terminus of the protein (Fig. 1A) (Kim *et al.*, 2006), it is possible that AIF may also be processed and released from the endoplasmic reticulum (ER) and enter the nucleus to perform its function. To test this hypothesis, the proteins that were isolated from wild-type flowers were used to perform a western blot analysis with an anti-AIF antibody. If the AIF protein was processed and released from the ER in young flower buds, only a cleaved 40kDa AIF-C would be detected by the anti-AIF antibody (Fig. 3A). As expected, this band was clearly obtained (Fig. 3B). Thus, as proposed, the AIF protein must be processed and released from the ER in the young flower buds of wild-type *Arabidopsis* to perform its function. To test further the specificity of the anti-AIF antibody and the processing of the AIF proteins, transgenic plants that ectopically expressed the full-length AIF were further generated and the proteins from these *35S:AIF+GFP* transgenic flowers were isolated in order to perform a western blot analysis using the anti-AIF antibody. Two bands, a 40kDa AIF-C (cleaved) and an 87kDa AIF+GFP (green fluorescent protein) fusion protein (uncleaved), were detected (Fig. 3A, B). The detection of the 40kDa AIF-C band demonstrates that the transgenic as well as endogenous AIF protein was processed and released from the ER in floral buds in the *35S:AIF+GFP* transgenic plants. The detection of the 87kDa AIF+GFP band indicates that some transgenic AIF proteins were most probably not processed and released from the ER in the mature flowers in the *35S:AIF+GFP* transgenic plants.

**Fig. 4.**
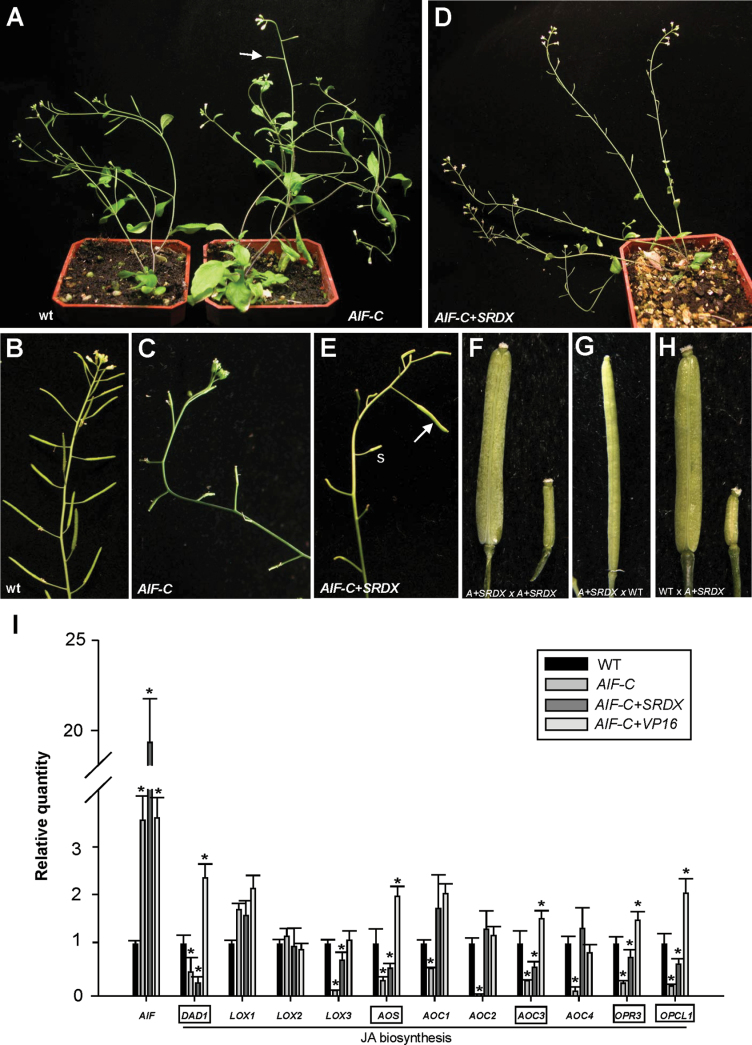
Phenotypic analysis of the *35S:AIF-C* and *AIF-C+SRDX Arabidopsis* plants and the detection of gene expression in *Arabidopsis* plants with altered *AIF* expression. (A) A 40-day-old *35S:AIF-C* plant (right) was sterile and produced short siliques (arrowed), whereas a wild-type plant (left) produced a well-developed, long silique. (B and C) The wild-type inflorescence (B) contained elongated siliques, whereas short and undeveloped siliques were observed in the *35S:AIF-C* plant (C). (D) A 50-day-old *AIF-C+SRDX* plant was sterile and produced short siliques. (E) The *AIF-C+SRDX* flower that was manually pollinated with *AIF-C+SRDX* pollen developed a well-elongated silique (arrowed), whereas short siliques (s) developed without manual pollination. (F) Close-up of a well-elongated silique (*AIF-C+SRDX×AIF-C+SRDX*) (left) and a short silique (right) from (E). (G) The emasculated wild-type flower that was pollinated with *AIF-C+SRDX* pollen developed an elongated silique. (H) The *AIF-C+SRDX* flower that was pollinated with wild-type pollen developed elongated siliques (left), whereas short siliques (right) developed in the absence of wild-type pollen pollination. (I) mRNA accumulation for *AIF*, *DAD1*, *LOX1*, *LOX2*, *LOX3*, *AOS*, *AOC1*, *AOC2*, *AOC3*, *AOC4*, *OPR3*, and *OPCL1* as determined by real-time quantitative RT–PCR. Total RNAs that were isolated from the flower buds before stage 12 of *35S:AIF-C* (*AIF-C*), *AIF-C+SRDX*, *AIF-C+VP16*, and wild-type Columbia (WT) plants were used as templates. The columns represent the relative expression of the genes. The genes showing clear down-regulation in the *35S:AIF-C* and *AIF-C+SRDX* plants and up-regulation in the *AIF-C+VP16* plants are boxed. The transcript levels of these genes were determined using 2–3 replicates and were normalized using *UBQ10*. The expression of each gene was relative to that in the wild-type plant, which was set at 1. The error bar represents the standard deviation. Each experiment was repeated twice with similar results. The asterisks indicate a significant difference from the wild-type value (*P < 0.05).

**Fig. 5.**
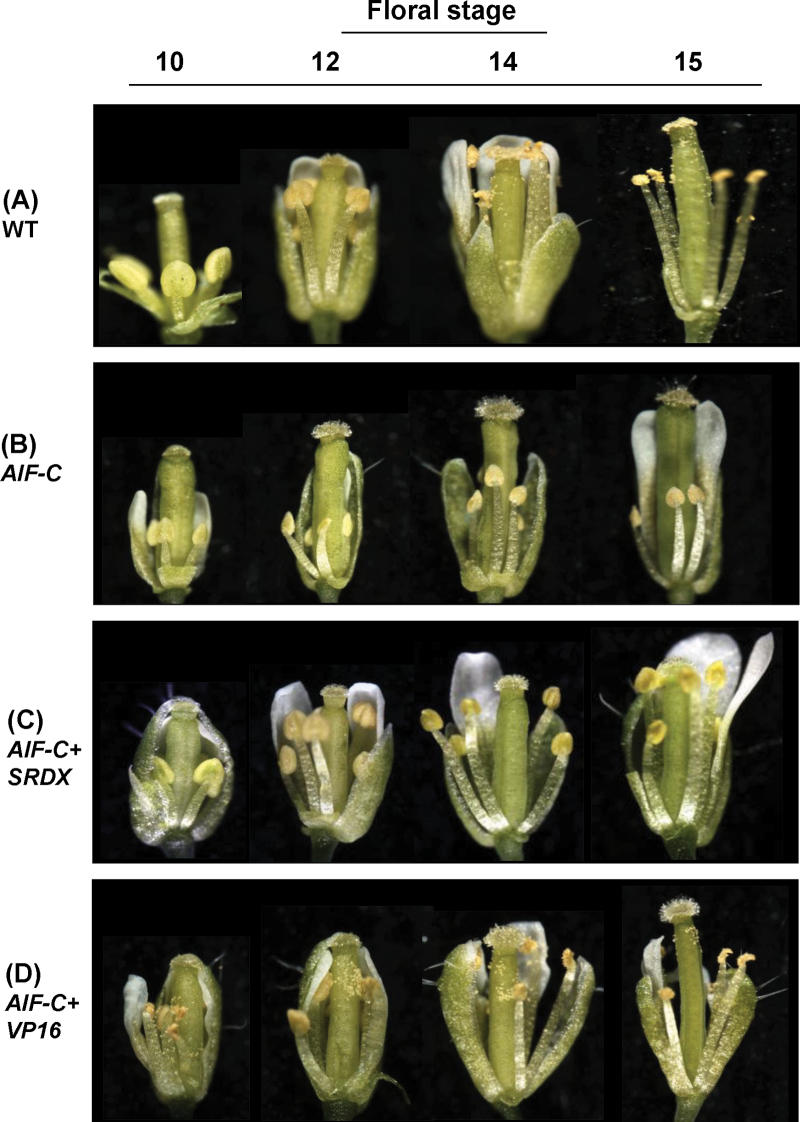
Phenotypic analysis of flowers in different development stages (10, 12, 14, and 15) for *Arabidopsis* with altered expression of *AIF.* (A) Wild-type flowers showed anther dehiscence after stage 12. (B and C) Anther dehiscence was not observed in *35S:AIF-C* (B) or *AIF-C+SRDX* (C) flowers even after stage 15. (D) *AIF-C+VP16* flowers showed early anther dehiscence at stage 10. The pollen of *AIF-C+VP16* flowers is apparently normal but unable to reach the stigmatic papillae of the carpel at the proper time.

**Fig. 6.**
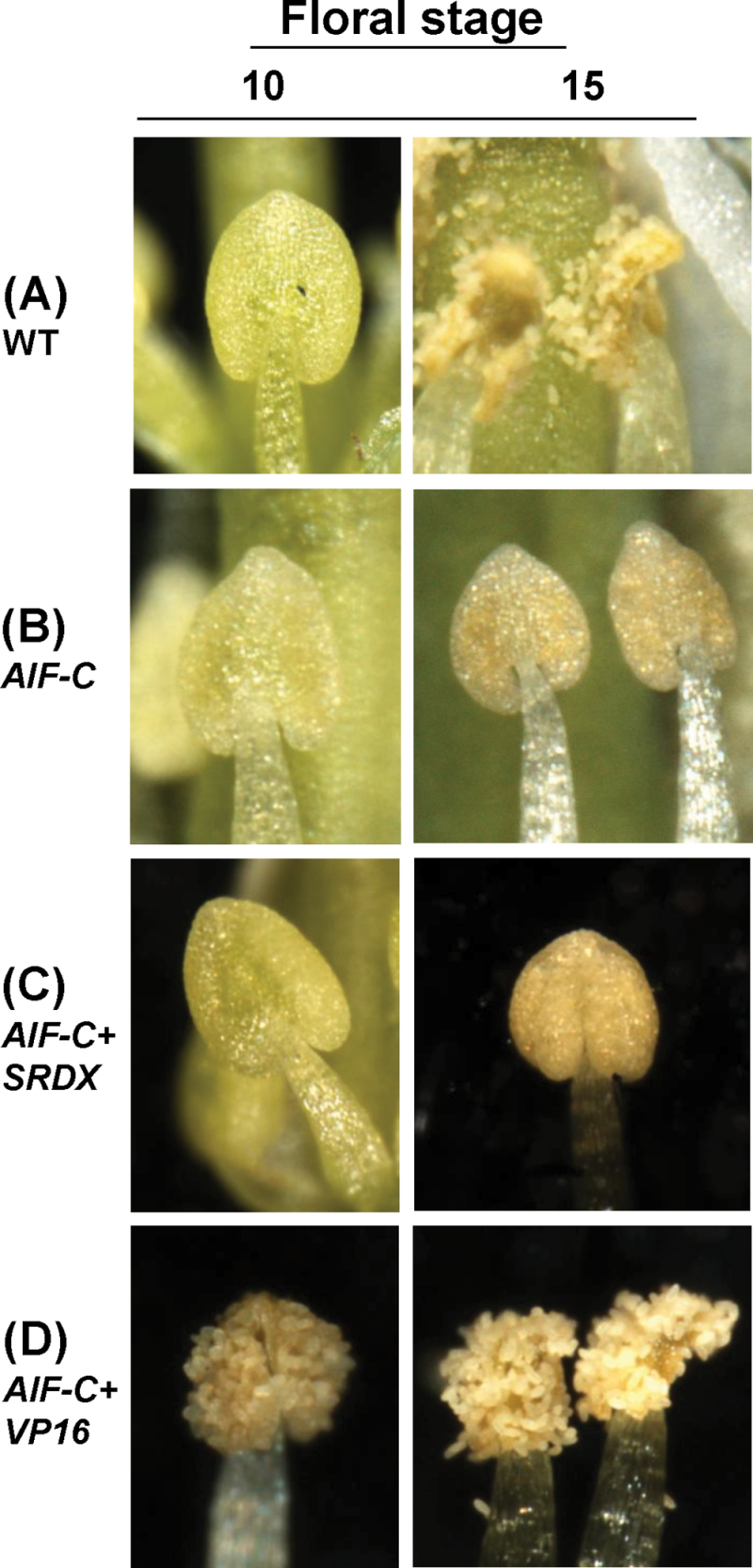
Phenotypic analysis of the anthers at stages 10 and 15 for *Arabidopsis* with altered expression of *AIF*. (A) In wild-type flowers, anthers were not dehiscent at stage 10. Anthers were completely dehiscent and the pollen was released at stage 15. (B and C) Anther dehiscence was not observed in *35S:AIF-C* (B) or *AIF-C+SRDX* (C) flowers at either stage 10 or stage 15. (D) In *AIF-C+VP16* flowers, anthers were dehiscent and the pollen was released at stage 10. Anthers were completely dehiscent at stage 15. (This figure is available in colour at *JXB* online.)

To test this hypothesis further, a strategy was adopted of transforming *YFP* fused with *AIF* (*35S:YFP+AIF*) or the *AIF* cDNA lacking its protein’s C-terminal region (*35S:YFP+AIF-C*) into *Arabidopsis* mesophyll protoplasts and analysing their fluorescence images. A comparison of the YFP fluorescence images of the nuclei showed that only the YFP+AIF-C fusion proteins accumulated in the nuclei of the cells (Fig. 3C-1, C-2). In contrast, the YFP+AIF fusion proteins accumulated in the membrane and were absent in the nuclei (Fig. 3C-3, C-4). As controls, YFP fluorescence images were dispersed in the cytoplasm in the *35S:YFP* transgenic *Arabidopsis* (Fig. 3C-5, C-6).

### Ectopic expression of *AIF-C* causes anther indehiscence, alters pollen development, and causes plant sterility

To investigate the function of the *AIF* gene, the cDNA lacking the C-terminal region of the *AIF* gene (Fig. 1A), driven by the CaMV 35S promoter (*35S:AIF-*C), was transformed into *Arabidopsis*.

A total of three *35S:AIF-C* transgenic *Arabidopsis* plants showing similar abnormal phenotypes were obtained. When the inflorescence was examined, a sterile flower phenotype was observed in these *35S:AIF-C* transgenic plants (Fig. 4A), with the siliques failing to elongate during late development (Fig. 4A, C). This phenotype was significantly different from that of the wild-type inflorescence, with its elongated and fully developed siliques (Fig. 4A, B). When the *35S:AIF-C* (Fig. 5B) flowers were examined, they opened normally and produced normal sepals, petals, and carpels with fully developed stigmatic papillae that were similar to those observed in wild-type flowers (Fig. 5A). The anthers of these *35S:AIF-C* (Figs 5B, 6B) flowers were indehiscent at all stages of flower development, whereas the wild-type anthers were dehiscent after stage 12 of flower development (Figs 5A, 6A). Because the anthers of the *35S:AIF-C* flowers were unable to open throughout flower development, these flowers were sterile and unable to set seed (Fig. 4A, C).

**Fig. 7.**
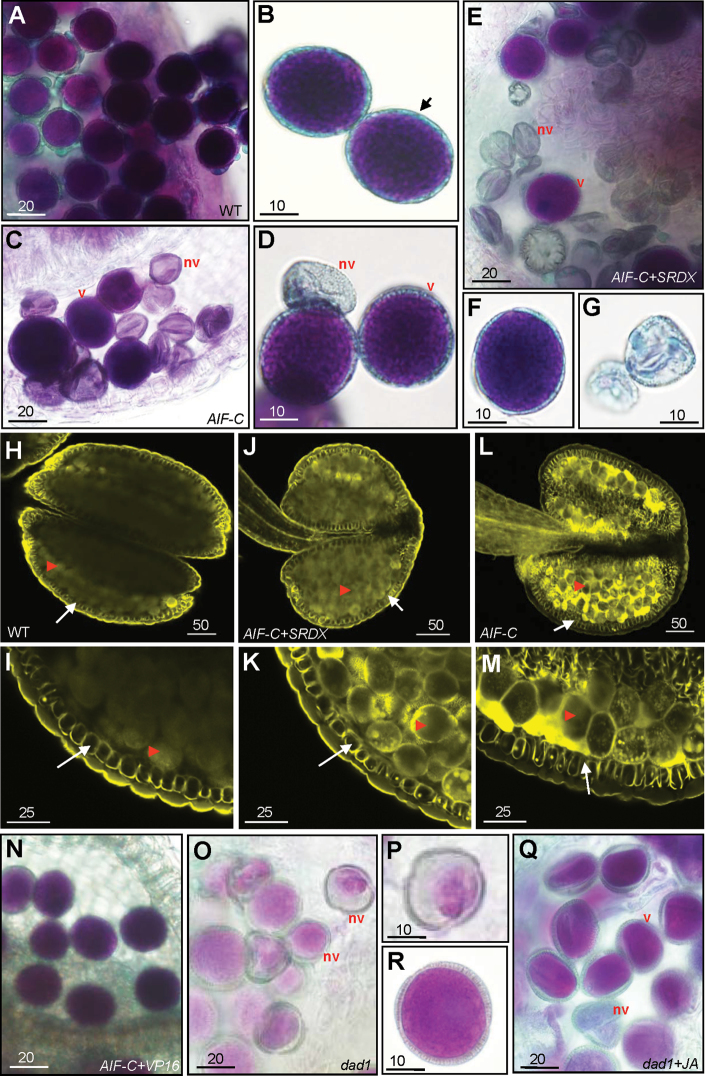
Alexander’s staining of the pollen and lignin staining of the anther in various *AIF* transgenic plants. (A) Pollen grains with normal viability were observed in the wild-type anther. (B) Close-up of the wild-type pollen with the thick outer wall (arrow). (C) Pollen grains with normal viability (v) or non-viability (nv) with defective small and collapsed shapes were observed in a *35S:AIF-C* anther that was manually forced to open. (D) Close-up of viable pollen grains (v) and non-viable pollen grains (nv) from a *35S:AIF-C* anther. (E) Pollen grains with normal viability (v) or non-viability (nv) with defective small and collapsed shapes were observed in an *AIF-C+SRDX* anther that was manually opened. (F and G) Close-up of viable pollen grains (F) and non-viable pollen grains (G) from an *AIF-C+SRDX* anther. (H–M) Anthers at stage 11, just prior to anther dehiscence, were stained with auramine O and observed by confocal microscopy (488nm excitation/510–560nm emission). Secondary thickening is visible in the endothecium (arrow) of the anthers of wild-type (H, I), *AIF-C+SRDX* (J, K), and *AIF-C* (L, M) plants. The arrowheads indicate the pollen grains. I, K, and M are close-up images of H, J, and L, respectively. (N) Pollen grains with normal viability were observed in the *AIF-C+VP16* anthers. (O) Non-viable pollen grains (nv) with defective small and collapsed shapes were observed in a *dad1* mutant anther. (P) Close-up of the non-viable defective pollen from (O). (Q) Pollen grains with normal viability (v) or non-viability (nv) were observed in a *dad1* mutant anther that was treated with JA. (R) Close-up of viable pollen from a JA-treated *dad1* mutant. Bar=20 μm in A, C, E, N, O, and Q; 10 μm in B, D, F, G, P, and R; 50 μm in H, J, and L; and 25 μm in I, K, and M.

**Fig. 8.**
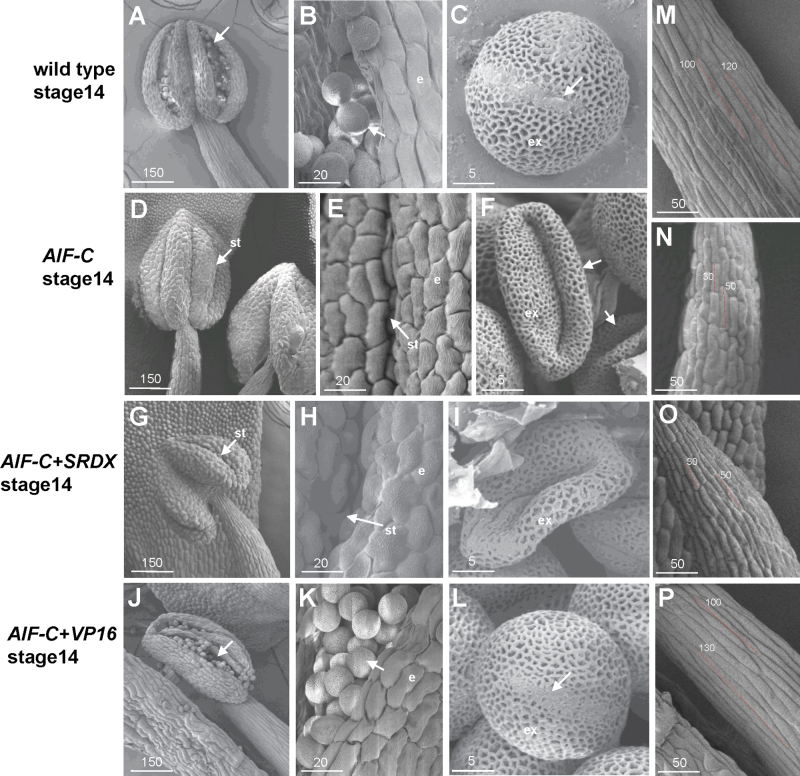
Scanning electron micrographs of pollen produced in various *AIF* transgenic plants. (A) Anther was dehiscent and pollen (arrowed) were released in a stage 14 wild-type flower. (B) Close-up of the egg-shaped wild-type pollen grains (arrowed) with a slightly triangular aspect from (A). e, epidermis. (C) Close-up of the wild-type pollen grains from (B). Colpi (arrowed) and outer exine (ex) with a typical irregular wall structure were observed on the surface of the pollen. (D) The anther was indehiscent and pollen were not released in a stage 14 *35S:AIF-C* flower. st, stomium. (E) Close-up of the indehiscent stomium (st) of the *35S:AIF-C* anther from (D). e, epidermis. (F) Close-up of severely collapsed pollen grains (arrowed) when the anther was opened manually from (D). ex, outer exine. (G) Anther was indehiscent and pollen were not released in a stage 14 *AIF-C+SRDX* flower. st, stomium. (H) Close-up of the indehiscent stomium (st) of the *AIF-C+SRDX* anther from (G). e, epidermis. (I) Close-up of a severely collapsed pollen grain when the anther was opened manually from (G). ex, outer exine. (J) Anther was dehiscent and wild-type-like pollen (arrowed) was released in a stage 14 *AIF-C+VP16* flower. (K) Close-up of the wild-type-like pollen grains (arrowed) from (J). e, epidermis. (L) Close-up of the wild-type-like pollen grains from (K). Colpi (arrowed) and outer exine (ex) were observed on the surface of the pollen. (M) Close-up of the epidermal cells (~100–120 μm in length) in the stamen filament of a mature wild-type flower from (A). (N) Close-up of the epidermal cells (~30–50 μm in length) in the stamen filament of a *35S:AIF-C* flower from (D), which are approximately three times shorter than those in (M). (O) Close-up of the epidermal cells (~30–50 μm in length) in the stamen filament of an *AIF-C+SRDX* flower from (G), which are approximately three times shorter than those in (M). (P) Close-up of the epidermal cells (~100–130 μm in length) in the stamen filament of an *AIF-C+VP16* flower from (J), which are similar to those in (M). Bar=150 μm in A, D, G, and J; 50 μm in M, N, O, and P; 20 μm in B, E, H, and K; and 5 μm in C, F, I, and L.

To examine pollen viability further, Alexander’s stain, which can distinguish viable pollen from non-viable pollen (Alexander, 1969), was applied. Normal viability (dark blue staining), similar to that of the wild-type pollen (Fig. 7A, B), was observed in the *AIF-C* pollen (Fig. 7C, D). This finding indicates that some of the pollen that was produced in the *AIF-C* flowers was still functional. However, non-viable pollen grains with small and collapsed shapes were also observed (Fig. 7C, D). When further examined by SEM, the pollen grains that were released from wild-type anthers exhibited an egg shape of 30×5 μm (Fig. 8A–C). When the indehiscent anthers (Fig. 8D, E) from the stage 14 flowers of the *AIF-C* plants were opened manually and their contents were compared with wild-type pollen, both abnormally collapsed pollen grains (Fig. 8F) and pollen grains normal in appearance were observed.

When the stamens were further examined in the *35S:AIF-C* (Fig. 5B) flowers, short stamen filaments were observed. When the epidermal cells in the stamen filaments were examined, the short stamens in the *35S:AIF-C* flowers were significantly reduced (~2- to 3-fold) in cell elongation (Fig. 8N) compared with that of the wild-type stamens (Fig. 8M). The lengths of the stamens in the *35S:AIF-C* (Fig. 5B) plants were also approximately one-third to a half the length of the wild-type stamens (Fig. 5A), indicating that the ectopic expression of *AIF-C* altered cell expansion rather than proliferation during filament development.

### Ectopic expression of *AIF-C+SRDX* causes similar anther indehiscence, alters pollen development, and causes plant sterility

To explore the role of *AIF* in regulating plant growth and development, its function was evaluated through a loss-of-function analysis. When T-DNA insertional mutants of *AIF* (Salk 018311) were analysed, they were phenotypically indistinguishable from wild-type plants in both vegetative and reproductive development. This finding indicates a possible functional redundancy between *AIF* and other genes.

To test this hypothesis, a strategy for generating transgenic dominant, loss-of-function mutant plants was employed by fusing a conserved SRDX-suppressing motif containing a 12 amino acid repressor sequence (LDLDLELRLGFA) to AIF-C (*AIF-C+SRDX*) (Fig. 1A). This strategy has been successfully used to generate dominant-negative mutants for studying the function of transcriptional activators with redundant functions (Hiratsu *et al.*, 2003; Eklund *et al.*, 2010; Guo *et al.*, 2010; Tejedor-Cano *et al.*, 2010).

A total of 10 *AIF-C+SRDX* transgenic plants with the mutant phenotype were obtained. Interestingly, an unexpected phenotype was observed in these *AIF-C+SRDX* transgenic *Arabidopsis* plants, anther indehiscence and flower sterility (Fig. 4D, E), which were similar to that of the *35S:AIF-C* flowers. When the *AIF-C+SRDX* flowers (Fig. 5C) were examined, their anthers (Figs 5C, 6C) were indehiscent at all stages of flower development, similar to those observed in the *35S:AIF-C* transgenic flowers (Figs 5B, 6B). Similar to the *35S:AIF-C* pollen, viable and non-viable pollen grains with small and collapsed shapes were observed in the *AIF-C+SRDX* flower by Alexander’s staining (Fig. 7E–G) and SEM (Fig. 8G–I). To examine further the viability of the pollen, mutant pollen grains were manually placed on the stigmas of mutant and wild-type flowers, and silique elongation and seed maturation were observed (Fig. 4E–G). The results confirmed that some of the pollen grains from the *AIF-C+SRDX* flowers were viable. The carpels of *AIF-C+SRDX* flowers were fertile because their siliques elongated and seeds were produced after pollination by wild-type pollen (Fig. 4H). Although the short filament phenotype was not clearly observed in some *AIF-C+SRDX* flowers (Fig. 5C), short stamen filaments that were significantly reduced (~2- to 3-fold) in cell elongation compared with the wild-type stamens (Fig. 8M) were also observed in some of the *AIF-C+SRDX* flowers (Fig. 8O) similar to those in the *35S:AIF-C* flowers (Fig. 8N).

The presence of a conserved NARD domain in AIF (Fig. 1A, B; Supplementary S1B at *JXB* online) indicates a possible transcriptional repressor role for AIF. Therefore, the overexpression of *AIF-C+SRDX* may cause a similar repression and result in a phenotype similar to that observed in the *35S:AIF-C* transgenic plants. The function of the SRDX-suppressing motif that was used in this study was confirmed by generating an *ag* mutant phenotype in transgenic *Arabidopsis* flowers that were transformed with the construct *AGAMOUS+SRDX* (*AG+SRDX*) (Chen *et al.*, 2011).

To analyse further the cellular basis for anther dehiscence, lignin staining with auramine O was performed in the endothecium of the developing anther in *AIF-C*, *AIF-C+SRDX*, and wild-type plants in order to examine the formation of the secondary wall thickness. In the wild-type plants, secondary thickening occurs in the endothecium before anther dehiscence, and the surrounding cell layers of the anther did not undergo secondary thickening (Yang *et al.*, 2007; Cecchetti *et al.*, 2013). In stage 11, just prior to anther dehiscence, similar structural features in the secondary thickening of cell walls in the endothecium, as well as pollen production, were observed in wild type (Fig. 7H, I), *AIF-C+SRDX* (Fig. 7J, K), and *AIF-C* (Fig. 7L, M) anthers. This result further supports the hypothesis that the developmental processes of the anther in *AIF-C+SRDX* and *AIF-C* plants proceed normally toward dehiscence but are interrupted instantly after septum/stomium breakage. This process is similar to that of the *dad1* mutant plants, which are altered in JA biosynthesis (Ishiguro *et al.*, 2001).

### Anther dehiscence was significantly promoted in *AIF-C+VP16* transgenic dominant-negative mutant plants

To investigate further the contrasting effects of the SRDX-suppressing motif, an activation domain, VP16-AD, which included an 11 amino acid activation sequence (DALDDFDLDML) was fused to AIF in order to produce *AIF-C+VP16* (Fig. 1A). This strategy has been used successfully to generate dominant-negative mutants for studying the function of transcriptional repressors with redundant functions (Ikeda *et al.*, 2009; Koo *et al.*, 2010; Chen *et al.*, 2011).

**Fig. 9.**
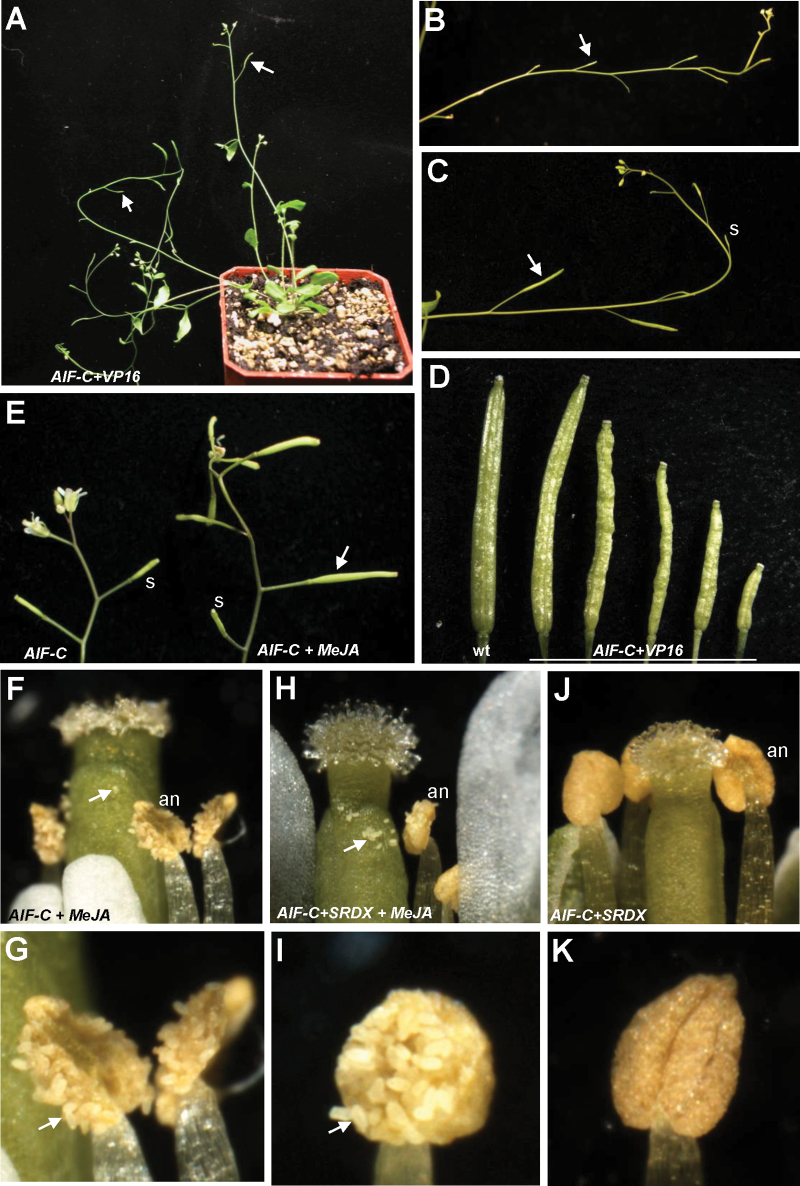
Phenotypic analysis of the *AIF-C+VP16 Arabidopsis* and the flowers of *35S:AIF-C* and *AIF-C+SRDX Arabidopsis* plants with and without JA treatment. (A) A 40-day-old *AIF-C+VP16* plants was sterile and produced short siliques (arrowed). (B) Close-up of the inflorescences of *AIF-C+VP16* that contained short and undeveloped siliques (arrowed). (C) Close-up of the inflorescences of *AIF-C+VP16* that contained short and undeveloped siliques (s) as well as siliques with incomplete elongation (arrowed). (D) A well-developed wild-type silique (left) and with five *AIF-C+VP16* siliques with different degrees of development and elongation. (E) The *35S:AIF-C* flower developed a well-elongated silique (arrowed on the right) after JA treatment, whereas undeveloped short siliques (s; left) were observed in *35S:AIF-C* flowers without JA treatment. (F) The anthers (an) were dehiscent and the pollen (arrowed) was released in a *35S:AIF-C* flower 2 d after JA treatment. (G) Close-up of the dehiscent anthers and the released pollen (arrowed) in (F). (H) The anthers (an) were dehiscent and the pollen (arrowed) was released in an *AIF-C+SRDX* flower 2 d after JA treatment. (I) Close-up of the dehiscent anthers and the released pollen (arrowed) in (H). (J and K) The anthers (an) were indehiscent in an *AIF-C+SRDX* flower 2 d after Tween-20 treatment.

A total of 17 *AIF-C+VP16* transgenic plants exhibiting the mutant phenotype were obtained. When the inflorescence was examined, a novel sterile flower phenotype was observed (Fig. 9A): the siliques failed to elongate during late development (Fig. 9B). The *AIF-C+VP16* flowers opened normally and produced normal sepals, petals, and carpels with fully developed stigmatic papillae (Fig. 5D). In contrast to the wild-type flowers, early anther dehiscence was observed in the *AIF-C+VP16* transgenic plants (Figs 5D, 6D). The stamens of the *AIF-C+VP16* flowers were prematurely dehiscent at early stage 10–12 (Figs 5D, 6D), whereas the wild-type anthers remained indehiscent at this stage (Figs 5A, 6A). Interestingly, the early anther dehiscence phenotype of the *AIF-C+VP16* flowers was completely opposite to that which was observed for the *35S:AIF-C* or *AIF-C+SRDX* flowers, which showed anther indehiscence (Fig. 5B, C). Because the early anther dehiscence of the *AIF-C+VP16* flowers prevented the pollen from reaching the stigmatic papillae of the carpel at the proper time (Fig. 5D), these flowers were sterile and unable to set seeds (Fig. 9B). However, different degrees of silique elongation were occasionally observed in these *AIF-C+VP16* flowers (Fig. 9C, D). This finding indicates that the pollen from the *AIF-C+VP16* flowers was able to function when it reached the stigmatic papillae. The viability of the *AIF-C+VP16* pollen was further confirmed by Alexander’s staining (Fig. 7N) and SEM (Fig. 8J–L). The *AIF-C+VP16* flowers produced stamen filaments with epidermal cells similar in length (Fig. 8P) to those of the wild-type filaments (Fig. 8M).

The function of the VP16-AD activation domain that was used in this study was confirmed by generating a *sup* mutant phenotype in transgenic *Arabidopsis* flowers that were transformed with the construct *SUPERMAN-DR+VP16* (*SUP-DR+VP16*) (Chen *et al.*, 2011).

### The expression of genes that participate in JA biosynthesis was down-regulated in *35S:AIF-C* and *AIF-C+SRDX* and up-regulated in *AIF-C+VP16* transgenic *Arabidopsis*

Mutations in genes that participate in JA biosynthesis cause a similar failure or delay of anther dehiscence, resulting in male sterility in *Arabidopsis* (Sanders *et al.*, 1999, 2000; Stintzi and Browse, 2000). Does the ectopic expression of *AIF-C* or *AIF-C+VP16* affect the expression of these genes in transgenic plants and result in the alteration of anther dehiscence? To answer this question, total RNA was extracted before stage 12 from the flower buds of *35S:AIF-C*, *AIF-C+SRDX*, and *AIF-C+VP16* transgenic plants, and the expression of genes that are involved in JA biosynthesis was analysed by real-time quantitative RT–PCR analysis.

As expected, the *AIF* mRNA was up-regulated in the flower buds before stage 12 in the *35S:AIF-C, AIF-C+SRDX* and *AIF-C+VP16* transgenic plants (Fig. 4I). Interestingly, the levels of the *DAD1*, *AOS*, *AOC3*, *OPR3*, and *OPCL1* transcripts were clearly down-regulated in the flowers of both *35S:AIF-C* and *AIF-C+SRDX* and were up-regulated in the flowers of *AIF-C+VP16* (Fig. 4I). Although the expression of *LOX3* was down-regulated in the flowers of both *35S:AIF-C* and *AIF-C+SRDX*, its expression was unaffected in the flowers of *AIF-C+VP16* (Fig. 4I). These results strongly suggest that the altered anther dehiscence in *35S:AIF-C*, *AIF-C+SRDX*, and *AIF-C+VP16* plants is correlated with the altered expression of some of the genes that participate in JA biosynthesis.

To explore the correlation between defective pollen and JA biosynthesis further, the pollen viability of the *dad1* mutant was examined using Alexander’s staining. When the indehiscent anthers of the *dad1* plants were opened manually, most of the *dad1* pollen grains were non-viable, with small sizes and abnormal shapes (Fig. 7O, P). This result was in agreement with the very low viability (~2%) of the pollen in the original characterization of the *dad1* mutant (Ishiguro *et al.*, 2001). Ishiguro *et al.* (2001) reported that an external supply of JA increased the pollen viability and rescued the sterility of the *dad1* flowers. When the *dad1* pollen grains were examined by Alexander’s staining after JA treatment, pollen with normal viability (dark red stain) (Fig. 7Q, R), as well as non-viable pollen grains with small sizes and collapsed shapes (Fig. 7Q), were observed, similar to what was observed in the *AIF-C* and *AIF-C+SRDX* pollen (Fig. 7C–G). This finding indicates that the defective pollen that is produced in the *AIF-C* and *AIF-C+SRDX* flowers is likely to be due to an alteration of JA signalling similar to that observed in the *dad1* mutants.

### External application of JA rescued anther indehiscence in *35S:AIF-C* and *AIF-C+SRDX* flowers

To examine whether an external supply of JA rescues the sterility of the *35S:AIF-C* or *AIF-C+SRDX* flowers similar to the JA-treated *dad1* flowers, JA was externally applied to the bud clusters of the *35S:AIF-C* and *AIF-C+SRDX* plants. Anther dehiscence (Fig. 9F–I) and elongated siliques (Fig. 9E, right) with seeds were observed in the JA-treated *35S:AIF-C* and *AIF-C+SRDX* flowers. This result was clearly different from the JA-untreated mock flowers, which lacked anther dehiscence (Fig. 9J, K) and silique elongation (Fig. 9E, left). This result indicates that pollen grains of the *35S:AIF-C* and *AIF-C+SRDX* flowers are fertile once the anthers are dehiscent in response to the external application of JA.

### Suppression of *AIF* expression causes early anther dehiscence and plant sterility

**Fig. 10.**
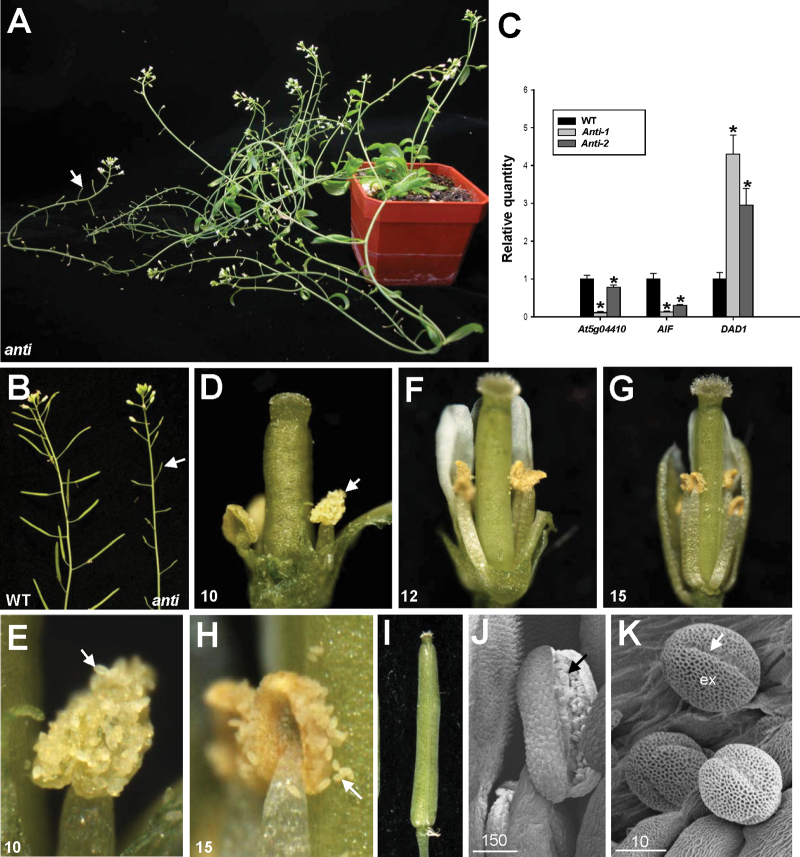
Phenotypic analysis of antisense plants with the suppression of *AIF* and *At5g04410* expression. (A) A 50-day-old *35S:AIF/At5g04410-*antisense plant was sterile and produced short siliques (arrowed). (B) Close-up of the inflorescences of antisense plant (right) that contained short and undeveloped siliques (arrowed), whereas wild-type plants (WT) produced long, well-developed siliques (left). (C) mRNA accumulation for *AIF*, *At5g04410*, and *DAD1* was determined by real-time quantitative RT–PCR. Total RNAs isolated from the floral buds before stage 12 of two *AIF/At5g04410* antisense (Anti-1, -2) and one wild-type Columbia (WT) plants were used as templates. Columns represent the relative expression of the genes. Transcript levels of these genes were determined using 2–3 replicates and were normalized using *UBQ10*. The expression of each gene was relative to that in the wild-type plant, which was set at 1. Error bar represents the standard deviation. Each experiment was repeated twice with similar results. For *At5g04410* expression: WT=1±0.094, Anti-1=0.11±0.024, Anti-2=0.78±0.058. For *AIF* expression: WT=1±0.143, Anti-1=0.13±0.015, Anti-2=0.30±0.022. For *DAD1* expression: WT=1±0.167, Anti-1=4.30±0.507, Anti-2=2.95±0.439. The asterisks indicate a significant difference from the wild-type value (*P < 0.05). (D) An *AIF/At5g04410* antisense flower showed early anther dehiscence (arrowed) at stage 10. (E) Close-up of the dehiscent anthers and the released pollen (arrowed) in (D). (F and G) Anther dehiscence was observed in *AIF/At5g04410* antisense flowers at stage 12 (F) and 15 (G). (H) Close-up of the dehiscent anthers and the released pollen (arrowed) in (G). (I) The emasculated wild-type flower pollinated with *AIF/At5g04410* antisense pollen developed an elongated silique. (J) Anther was dehiscent and wild-type-like pollen (arrowed) was released in a stage 10 *AIF/At5g04410* antisense flower. Bar=150 μm. (K) Close-up of the wild-type-like pollen grains from (J). Colpi (arrowed) and outer exine (ex) were observed on the surface of the pollen. Bar=10 μm.

Because *AIF* may be functionally redundant with other genes, a loss-of-function analysis using an antisense strategy with DNA fragments, including a highly conserved NAC domain for *AIF* and its most closely related gene, At5g04410 (Supplementary Fig. S1 at *JXB* online), was performed. It was expected that *AIF* and potentially redundant genes such as At5g04410 would be suppressed. A total of five *35S:AIF/At5g04410* antisense transgenic plants exhibiting the mutant phenotype were obtained (Fig. 10A, B). As expected, a clear reduction of *AIF* and *At5g04410* expression was observed in the antisense plants (Fig. 10C). This result indicates that the phenotypes of these antisense *Arabidopsis* plants result from the simultaneous down-regulation of both *AIF* and the putative redundant genes. The vegetative leaf development of these transgenic plants was apparently normal. When the inflorescence was examined in these antisense plants, sterile flowers were observed (Fig. 10A, B); the siliques failed to elongate during late development (Fig. 10B). When the antisense flowers were examined, they opened normally and produced normal flower organs (Fig. 10D–H). In contrast to wild-type flowers, early anther dehiscence was observed in the antisense plants (Fig. 10D, E). The short stamens of the antisense flowers were prematurely dehiscent at early stage 10 (Fig. 10D, E), similar to the stamens of the *AIF-C+VP16* transgenic flowers (Figs 5D, 6D) and completely opposite to those of the *35S:AIF-C* and *AIF-C+SRDX* flowers, which showed indehiscence of the anthers (Fig. 5B, C). Because the short stamens of the antisense flowers were unable to reach the stigmatic papillae of the carpel (Fig. 10D, F, G), these flowers were sterile and unable to set seeds (Fig. 10A, B).

To examine the viability of the pollen further, the antisense pollen grains were manually placed on the stigmas of wild-type flowers, and silique elongation was observed (Fig. 10I). This finding indicates that the pollen from the antisense flowers was able to function when it reached the stigmatic papillae. The viability of the antisense pollen was further confirmed by SEM (Fig. 10J, K). Furthermore, the level of the *DAD1* transcripts was also up-regulated in the flower buds of the antisense plants before stage 12 (Fig. 10C), similar to what occurred in the *AIF-C+VP16* flowers. Interestingly, the increase in the *DAD1* transcripts correlated with the decrease in the *AIF* transcripts (Fig. 10C).

## Discussion

In this study, one gene, *AIF*, containing a NAC domain with an unknown function, was identified from and characterized in *Arabidopsis*. The ectopic expression of *AIF-C* caused a male-sterile phenotype with indehiscent anthers throughout flower development. It was therefore surmised that the function of the *AIF* gene is related to the regulation of anther dehiscence.

**Fig. 11.**
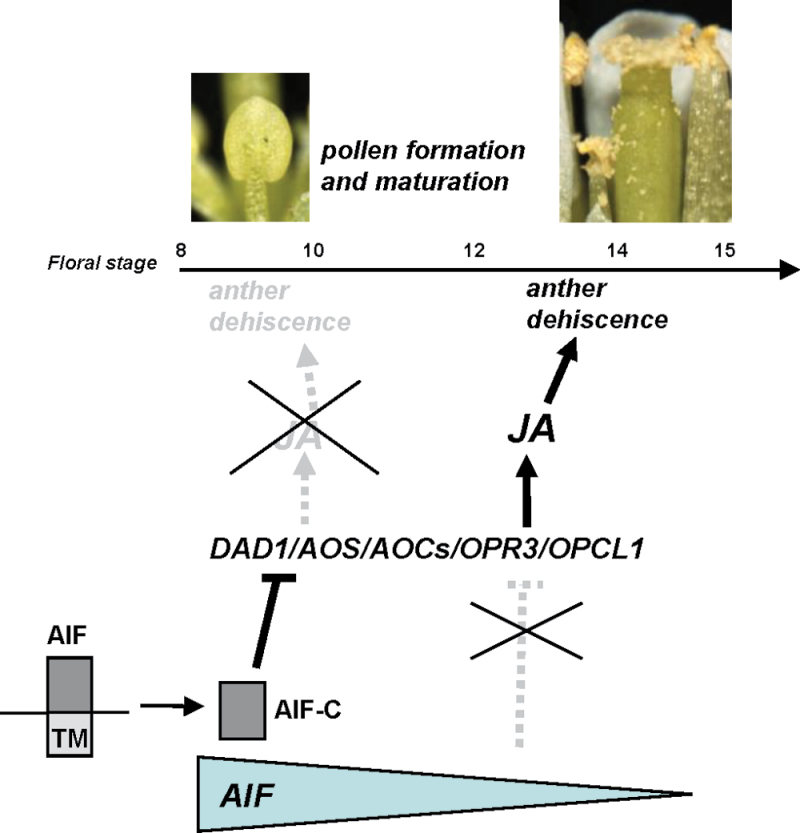
Model for the function of *AIF* in regulating stamen development in *Arabidopsis*. The gradient of *AIF* activity is illustrated by the gradual reduction in the size of the grey triangle during flower maturation. In wild-type *Arabidopsis*, the high expression of *AIF* and processing and release of AIF-C from the ER suppressed (—|) the expression of genes that participate in JA biosynthesis, such as *DAD1/AOS/AOCs*/*OPR3/OPCL1*, and the JA production in anthers during early flower development and resulted in the inhibition of anther dehiscence. During late flower development, the decrease of the *AIF* expression to a threshold level activated the expression of *DAD1/AOS/AOCs*/*OPR3*/*OPCL1* and increased (→) the JA production in the stamen. This caused the initiation (→) of anther dehiscence and resulted in the release of the normal mature pollen of wild-type flowers. In *AIF-C+VP16* or antisense plants, the expression of *DAD1/AOS/AOCs*/*OPR3*/*OPCL1* in stamen was activated during early flower development and resulted in a high level of JA production and ultimately caused early anther dehiscence. In contrast, in *35S:AIF-C* and *AIF-C+SRDX* plants, the expression of *DAD1/AOS/AOCs*/*OPR3*/*OPCL1* and the production of JA were suppressed by the high expression of AIF-C during all stages of flower development. This suppression prevented anther dehiscence and the release of pollen throughout flower development. The dashed (- - -) line/arrow indicates the pathways not activated in wild-type flowers. (This figure is available in colour at *JXB* online.)

This hypothesis was supported by the expression pattern of the *AIF* gene during flower development. GUS activity was detected in the anthers, the upper part of the filaments, and the pollen of the stamen of the *AIF:GUS* flower buds at stages 7–11 and was significantly decreased in mature flowers after stage 12 (Fig. 2). It is reasonable to suggest that the function of the *AIF* gene is to inhibit anther dehiscence during the early stages of flower development. When *AIF* expression decreased after maturation, this inhibition no longer occurred, thereby permitting anther dehiscence (Fig. 11). The identification of a conserved repressor NARD domain in AIF, as well as the similar deficiency in anther dehiscence in *AIF-C+SRDX* transgenic plants, indicates that *AIF* acts as a repressor controlling anther dehiscence. Thus, the ectopic expression of *AIF* in *35S:AIF-*C and *AIF-C+SRDX* plants extends its influence into the late stages of flower development and results in anther dehiscence inhibition throughout flower development.

*35S:AIF-C* plants (with or without +SRDX) produce the indehiscence phenotype. Clearly, AIF needs to be processed and released from the ER and must enter the nucleus to perform its function. This conclusion was supported by the finding that the full-length AIF protein is not able to enter the nucleus, whereas the AIF-C protein (without the transmembrane domain) can. The fact that only the cleaved AIF-C protein was detected in wild-type flowers using an anti-AIF antibody indicates that AIF is processed and released from the ER in young flower buds. In the *35S:AIF-C* and *AIF-C+SRDX* transgenic plants, the AIF-C protein that lacked the transmembrane domain could constitutively enter the nucleus during all stages of flower development, resulting in the constitutive suppression of anther dehiscence.

The function of *AIF* was further revealed by the analysis of *AIF-C+VP16* dominant-negative mutations. Interestingly, the anthers of the *AIF-C+VP16* flowers were prematurely dehiscent at early stage 10. This early-dehiscent phenotype was opposite to that of the *35S:AIF-*C and *AIF-C+SRDX* flowers, which showed anther indehiscence. Furthermore, a similar early-dehiscent phenotype was also observed in the loss-of-function mutants that were generated by an antisense strategy in which *AIF* and putatively redundant genes were simultaneously suppressed. These results further support the assumption that *AIF* functions as a transcriptional repressor to prevent anther dehiscence during the early stages of floral development (Fig. 11). When the function of the *AIF* gene was altered in the *AIF-C+VP16* mutants, this inhibition was reversed, resulting in significantly earlier anther dehiscence. Furthermore, this result also indicates that the VP16 activation domain that was used in this study is sufficient to overcome the NARD repressor domain in *AIF-C+VP16* transgenic plants.

One interesting and critical task is to determine the exact role of *AIF* in the negative regulation of anther dehiscence. The phenotype of the indehiscent anthers in *35S:AIF-C* and *AIF-C+SRDX* flowers resembled that of plants with mutations in genes that participate in JA biosynthesis (Sanders *et al.*, 2000; Ishiguro *et al.*, 2001; Scott *et al.*, 2004). Interestingly, the transcript levels of several genes that participate in JA biosynthesis, such as *DAD1/AOS/AOC3/OPR3/OPCL1*, were down-regulated in *35S:AIF-C* and *AIF-C+SRDX* plants. Thus, these genes are likely to function downstream of *AIF*. This hypothesis was further supported by the up-regulation of these genes (*DAD1/AOS/AOC3/OPR3/OPCL1*) in *AIF-C+VP16* and antisense mutants, in which anther dehiscence was promoted. These results indicate that *AIF* probably controls anther dehiscence by negatively regulating both the expression of genes that participate in JA biosynthesis and the level of JA. This hypothesis was further supported by two lines of evidence. First, similarities were observed between the *35S:AIF-C* and *AIF-C+SRDX* plants and the *dad1* mutants as follows: defective pollen, short filaments, and non-dehiscent anthers. The *35S:AIF-C* and *AIF-C+SRDX* plants appeared to produce more viable pollen than did the *dad1* mutants. In addition, filament elongation seems not to be completely affected in the *AIF-C* or *AIF-C+SRDX* flowers. The short filament was indeed produced in some (Fig. 8O) but not all *AIF-C+SRDX* flowers (Fig. 5C). These results were probably due to the incomplete down-regulation of *DAD1* and other genes that participate in JA biosynthesis in *35S:AIF-C* and *AIF-C+SRDX* plants. Thus, not all of the JA-regulated processes in the flowers will be completely affected in the *AIF-C* and *AIF-C+SRDX* flowers, as seen in the present results. Secondly, the external application of JA not only rescued the non-dehiscence of the anthers but also restored the defects of the pollen and caused the elongation of the silique and the production of seeds in the *35S:AIF-C* and *AIF-C+SRDX* flowers. These results were similar to those observed in the *dad1* mutant flowers (Ishiguro *et al.*, 2001).

The present results reveal a possible model for the interaction of *AIF* and JA in regulating anther dehiscence in *Arabidopsis*, as illustrated in Fig. 11. In wild-type *Arabidopsis*, a high level of *AIF* expression and the constitutive processing and release of AIF-C from the ER in the anthers and the upper part of the filaments during early stamen development suppressed *DAD1/AOS/AOC3/OPR3*/*OPCL1* gene expression and JA production and prevented early anther dehiscence. During late flower development, *AIF* expression was significantly reduced and was not sufficient to suppress *DAD1/AOS/AOC3/OPR3*/*OPCL1* expression. Thus, these genes were activated, and JA production was increased in the stamen, resulting in the initiation of anther dehiscence and the release of mature pollen. In the *AIF-C+VP16* dominant-negative mutant or antisense plants, *AIF* was either converted into an activator or suppressed during early flower development, resulting in the early activation of these genes and a high level of JA production in the stamens. Thus, early anther dehiscence occurred, and pollen was released. In contrast, in the *35S:AIF-C* and *AIF-C+SRDX* plants, the expression of *DAD1/AOS/AOC3/OPR3/OPCL1* and the production of JA were suppressed due to the high level of *AIF-C* or *AIF-C+SRDX* expression during all stages of flower development. The suppression of these genes caused anther indehiscence throughout flower development, similar to that observed in the *dad1* and *opr3* mutants.

In addition to *AIF*, it has been previously reported that several *NAC*-like genes are also involved in the regulation of male sterility in plants. For example, double mutants of two *NAC*-like genes, *NAC SECONDARY WALL THICKENING PROMOTING FACTOR 1* (*NST1*) and *NST2*, caused male sterility with a similar anther-indehiscent phenotype to that of *35S:AIF-C* and *AIF-C+SRDX* plants (Mitsuda *et al.*, 2005). However, *NST1* and *NST2* regulate secondary wall thickening in various tissues, and the anther dehiscence that was observed in the *nst1/nst2* double mutants was due to the loss of secondary wall thickening in the anther endothecium (Mitsuda *et al.*, 2005), which is completely different from the *AIF*-regulated mechanism. Transgenic rice plants ectopically expressing an RNAi (RNA interference) construct targeting the rice *NAC*-like gene *Os07g37920* produced reduced proportions of viable pollen grains and did not undergo anther dehiscence (Distelfeld *et al.*, 2012). This result indicates that the function of *Os07g37920* is the promotion of anther dehiscence, which is opposite to that of *AIF*. The precise molecular mechanisms regulated by *Os07g37920* during anther dehiscence remain to be elucidated. Thus, the unique characteristics of *AIF* in regulating anther dehiscence and JA biosynthesis found in this study provide useful information for understanding the additional functions of the *NAC*-like genes in regulating anther dehiscence and male sterility in plants.

In summary, one *NAC*-like gene, *AIF*, was characterized in *Arabidopsis*. The possible function of *AIF* in regulating the early development of the anther is supported by the early anther dehiscence in *AIF-C+VP16* transgenic dominant-negative mutant and loss-of-function antisense plants and by anther indehiscence in *35S:AIF-C* and *AIF-C+SRDX* flowers. This result indicates the importance of *AIF* in preventing anther dehiscence during early flower development. Furthermore, the present data provide evidence for a mechanism that functions upstream of JA signalling during anther dehiscence. In this mechanism, it was found that *AIF* needs to be processed and released from the ER to regulate anther dehiscence negatively by suppressing genes that participate in JA biosynthesis.

## Supplementary data

Supplementary data are available at *JXB* online.

Figure S1. Gene structure and protein sequence of AIF.

Figure S2. Phylogenetic analysis of selected *Arabidopsis NAC*-like genes.

Supplementary Data
