# Supplementary material for: The NAC-like gene ANTHER INDEHISCENCE FACTOR acts as a repressor that controls anther dehiscence by regulating genes in the jasmonate biosynthesis pathway in Arabidopsis
Source: J Exp Bot. 2013 Dec 9;65(2):621–39. doi: 10.1093/jxb/ert412 (PMC3904717; doi:10.1093/jxb/ert412)
Supplement: Supplementary Data [file supp_65_2_621__index.html]

The NAC-like gene ANTHER INDEHISCENCE FACTOR acts as a repressor that controls anther dehiscence by regulating genes in the jasmonate biosynthesis pathway in Arabidopsis — The NAC-like gene ANTHER INDEHISCENCE FACTOR acts as a repressor that controls anther dehiscence by regulating genes in the jasmonate biosynthesis pathway in Arabidopsis — Supplementary Data 

# The *NAC*-like gene *ANTHER INDEHISCENCE FACTOR* acts as a repressor that controls anther dehiscence by regulating genes in the jasmonate biosynthesis pathway in *Arabidopsis*

## Supplementary Data

Data files

**Files in this Data Supplement:**

- Supplementary Data - Supplementary Data
