# Supplementary material for: The NAC-like gene ANTHER INDEHISCENCE FACTOR acts as a repressor that controls anther dehiscence by regulating genes in the jasmonate biosynthesis pathway in Arabidopsis
Source: J Exp Bot. 2013 Dec 9;65(2):621–39. doi: 10.1093/jxb/ert412 (PMC3904717; doi:10.1093/jxb/ert412)
Supplement: Supplementary Data [file supp_ert412_jexbot097279_file001.pdf]

Supplementary Table S1. Oligo nucleotide sequence of primers used in gene cloning and PCR analysis.

**Primer name**	**Primer sequences**
AtNAC-3-1	**TCTAGA**GCTCTTTTAAGTTATTCATAAAC
AtNAC-3-2	**TCTAGA**CATCTATATCAATGTACAAACAC
AtNACL3 5’	**TCTAGA**ATGGGTCGTGGCTCAGTAACAT
NACL3 3’ΔC-ter	**TCTAGA**AGATTGCAAAGTTTCCGAGTGCTT
pAtNACL3 5’BamHI	**GGATCC**ATGGAAGAGGAATGGGCTGATG
pAtNACL3 3’BamHI	**GGATCC**ATACTCGATGGACCAAAACTCAAAC
AtNACL3 5’-2	**TCTAGA**ATGGGTCGTGGCTCAGTAACATC
AtNACL3 (delC) 3’	**TCTAGA**GTTGTCAGAAGATTGCAAAGTTTCCGA
SRDX-for	ACTTCGCCTCGGCTTTGCGTGACATGGTAGATCTGACTAGT
SRDX-forII	**GGTACC**CTCGATCTTGATCTCGAACTTCGCCTCGGCTTTGCG
mGFP-revII	**GAGCTC**TTCATATGATCTGGGTATCTTG
VP16-for	**GGTACC**GCTCCACCTACCGATGTCAGCCTGGGGGACGA
VP16-rev	**GAGCTC**TACCCACCGTACTCGTCAATTC
VP16-forII	ATGTCAGCCTGGGGGACGAGCTTCACTTAGACG
AtNACL3(-TGA) 3’	**TCTAGA**CCTGGAAGAGACCAAAATGCTC
AIF realtime for-2	AGTGGGAGGAGGAGGATGATATG
AIF realtime rev-2	GATTCTGCTGGTTGCTCAAAGTAG
AtNACL3 5’X*ma*I	**CCCGGG**GGTCGTGGCTCAGTAACATCGCT
AtNACL3 3’X*ma*I	**CCCGGG**TCACCTGGAAGAGACCAAAATGCTCA
AtNACL3-C 3’X*ma*I	**CCCGGG**TCAGTTGTCAGAAGATTGCAAAGTTTCCGA
At5g04410 realtime for-	TGTGTTTATGGGTTCTCCTACTCTC
At5g04410 realtime rev-	AGACCAATGATTCCAACAATAACGG
DAD1 realtime for-	AGATCGCAAGGCTACTCCAATC
DAD1 realtime rev-	CCACGGCGTCTCCTCCAC
real-LOX1-For.	ACTCTTCGTCTTGTAAGCTCTG

real-LOX1-Rev.	GTGACCTTGAAAGCGGATTCG
real-LOX2-For.	ATGGAGAGTTGGAAGAGTGAGC
real-LOX2-Rev.	TCCACCATTGATAGGTCACTGC
real-LOX3-For.	TTCGCACTTGGTCCTGTTCAC
real-LOX3-Rev.	ATCCGCTTGGTGTCTCATTCG
real-AOS-For.	CGGGCGGGTCATCAAGTTC
real-AOS-Rev.	GCTCCCATCGTGAGTTCTCC
real-AOC1-For.	CCCAGACCAAGCAAAGTTCAAG
real-AOC1-Rev.	TCTCCGAGACCAAACCTAAAGC
real-AOC2-For.	ATCACTCACCACATAAAGTAAAAGTCTC
real-AOC2-Rev.	CGATGAAATTGTTGATTACATGAAAGATTG
real-AOC4-For.	AATGTGTCCCGTCCCTATAAGC
real-AOC4-Rev.	GCCACGAGCCTACTAAGCC
real-OPR3-For.	TGGACGCAACTGATTCTGACC
real-OPR3-Rev.	TGGTAGCGAGGTTGTGTAACG
real-OPCL1-For.	TTTATTGTTTGTGCTGTAGACCTGTAG
real-OPCL1-Rev.	GCCATTTATGTGTGTAATCTTCTGTG
RT-UBQ10-F	CTCAGGCTCCGTGGTGGTATG
RT-UBQ10-4-2	GTGATAGTTTTCCCAGTCAACGTC

Shih_Fig. S1

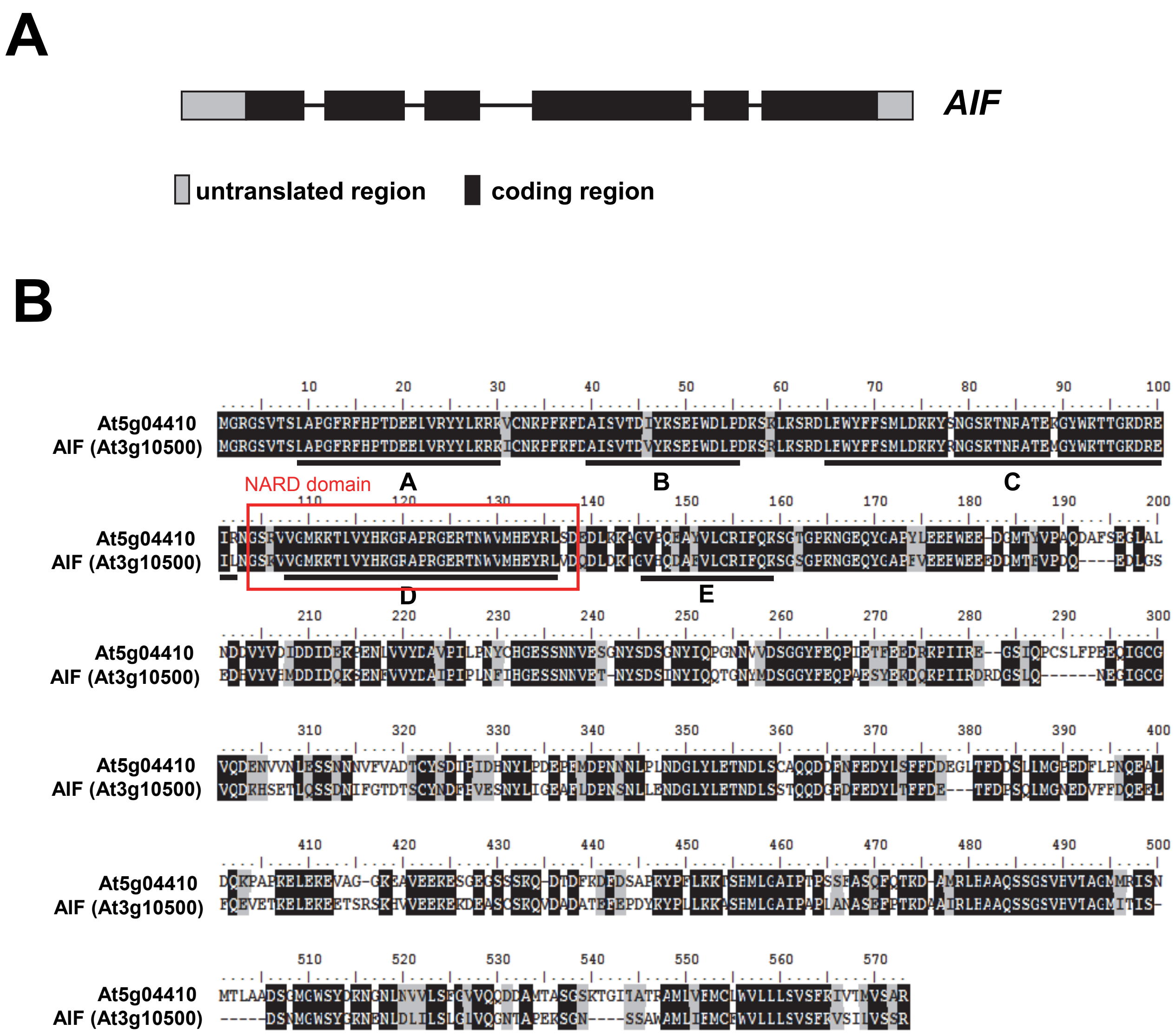

Supplementary Fig. S1 Gene structure and protein sequence of AIF.
(A) Gene structure of *AIF* (At3g10500), containing 6 exons (black bar) and 5 introns (line). 3' and 5' untranslated regions are indicated by a gray bar.
(B) Amino acid sequence alignment for AIF and At5g04410. The 5 conserved sub-domains (A-E) located in the NAC domain are shown with horizontal bars. The conserved NARD domain for repression of transcriptional activation in the end of the NAC domain was boxed in red color. In each alignment, identical or similar amino acid residues are indicated by black or gray boxes, respectively. To improve alignment, dashes were introduced into the sequence. This sequence alignment was generated by the ClustalW-Multiple Sequence Alignment Program at Biology Work Bench (http://workbench.sdsc.edu).

Shih_Fig. S2

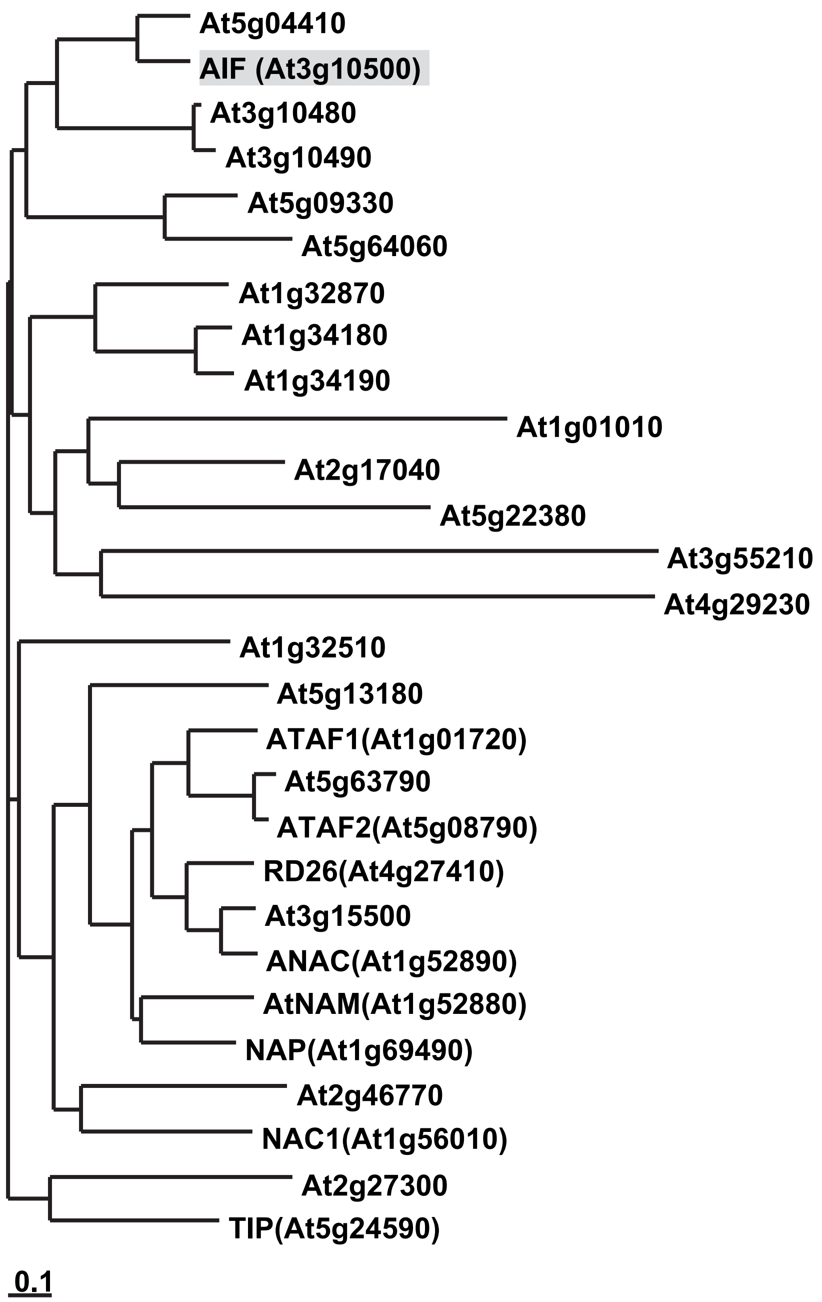

Supplementary Fig. S2 Phylogenetic analysis for selected *Arabidopsis NAC*-like genes. On the basis of amino acid sequence identity, AIF (At3g10500) is closely related to At5g04410. Name of the AIF protein is in gray boxes. The tree was generated by the Neighbor Joining (NJ) method, and the distance was calculated based on the Dayhoff PAM (Percent Allowed Mutation) matrix (Dayhoff *et al.,* 1983) using the PROTDIST program in PHYLIP software package (v3.5c, Kimura, 1980). Numbers on major branches indicate bootstrap percentages for 1,000 replicate analyses.
